# Lignin-Based Nonviral Gene Carriers Functionalized by Poly[2-(Dimethylamino)ethyl Methacrylate]: Effect of Grafting Degree and Cationic Chain Length on Transfection Efficiency

**DOI:** 10.3390/biom12010102

**Published:** 2022-01-08

**Authors:** Xiaohong Liu, Hui Yin, Xia Song, Zhongxing Zhang, Jun Li

**Affiliations:** 1 Department of Biomedical Engineering, National University of Singapore, Singapore 119276, Singapore; xiaohong.liu@nusricq.cn (X.L.); yin_hui@sp.edu.sg (H.Y.); a0045788@u.nus.edu (X.S.); 2 National University of Singapore (Chongqing) Research Institute, Chongqing 401120, China

**Keywords:** lignin, PDMAEMA, atom transfer radical polymerization, graft copolymer, plasmid DNA, nonviral gene carriers

## Abstract

Lignin is a natural renewable biomass resource with great potential for applications, while its development into high value-added molecules or materials is rare. The development of biomass lignin as potential nonviral gene delivery carriers was initiated by our group through the “grafting-from” approach. Firstly, the lignin was modified into macroinitiator using 2-bromoisobutyryl bromide. Then cationic polymer chains of poly[2-(dimethylamino)ethyl methacrylate] (PDMAEMA) were grown from the lignin backbone using atom transfer radical polymerization (ATRP) to yield lignin-PDMAEMA graft copolymers (LPs) with branched structure. To gain a deep understanding of the relationship between the nonviral gene transfection efficiency of such copolymers and their structural and compositional factors, herein eight lignin-based macroinitiators with different modification degrees (MDs, from 3.0 to 100%) were synthesized. Initiated by them, a series of 20 LPs were synthesized with varied structural factors such as grafting degree (GD, which is equal to MD, determining the cationic chain number per lignin macromolecule), cationic chain length (represented by number of repeating DMAEMA units per grafted arm or degree of polymerization, DP) as well as the content of N element (N%) which is due to the grafted PDMAEMA chains and proportional to molecular weight of the LPs. The in vitro gene transfection capability of these graft copolymers was evaluated by luciferase assay in HeLa, COS7 and MDA-MB-231cell lines. Generally, the copolymers LP-12 (N% = 7.28, MD = 36.7%, DP = 13.6) and LP-14 (N% = 6.05, MD = 44.4%, DP = 5.5) showed good gene transfection capabilities in the cell lines tested. Overall, the performance of LP-12 was the best among all the LPs in the three cell lines at the N/P ratios from 10 to 30, which was usually several times higher than PEI standard. However, in MDA-MB-231 at N/P ratio of 30, LP-14 showed the best gene transfection performance among all the LPs. Its gene transfection efficiency was ca. 11 times higher than PEI standard at this N/P ratio. This work demonstrated that, although the content of N element (N%) which is due to the grafted PDMAEMA chains primarily determines the gene transfection efficiency of the LPs, it is not the only factor in explaining the performance of such copolymers with the branched structure. Structural factors of these copolymers such as grafting degree and cationic chain length could have a profound effect on the copolymer performance on gene transfection efficiency. Through carefully adjusting these factors, the gene transfection efficiency of the LPs could be modulated and optimized for different cell lines, which could make this new type of biomass-based biomaterial an attractive choice for various gene delivery applications.

## 1. Introduction

The development of biomass resources into materials with high added value, which are renewable, cheap and easy to obtain, is of great importance to alleviate the problems of resources, such as energy crises and environmental pollution. Lignin is the second most abundant renewable biomass resource in nature and is the only renewable aromatic polymer containing a benzene ring [1,2]. However, due to the characteristics of lignin’s complex structure, broad polydispersity and limited chemical functional groups, only 1% of the total lignin in the industry is converted into valuable industrial products, while among them the conversion of lignin into high value-added products is quite limited [3]. During recent years, a lot of research interest on lignin towards high value-added applications has been aroused, especially in biomedical fields such as drug delivery and tissue engineering etc. due to the desired biocompatible, antioxidant and antimicrobial properties of lignin [4,5,6,7].

Gene therapy is a revolutionary biological therapy method, and gene delivery is one of the key factors to determine the effect of gene therapy [8]. Cationic polymer is an important kind of nonviral gene transfer vector, which exhibits no immunogenicity, carcinogenicity, good stability and is easy to synthesize, and therefore it has been greatly developed in recent decades [9,10,11,12,13,14,15,16,17]. Our group started to explore the feasibility to functionalize lignin for nonviral gene delivery, where kraft lignin was grafted with cationic polymer blocks through a “grafting-from” approach [18]. The process includes the modification of lignin into a lignin-based macroinitiator, followed by the grafting of poly[2-(dimethylamino)ethyl methacrylate] (PDMAEMA) to the lignin molecules through atom transfer radical polymerization (ATRP). By using this approach, multiple cationic PDMAEMA arms were attached to lignin backbone, efficiently yielding lignin-PDMAEMA graft copolymers with branched structure (LPs). The cationic arm number per lignin macromolecule (related to grafting degree) and arm length are controllable. The gene delivery vector based on such lignin graft copolymer was proven to be less cytotoxic. Compared with branched PEI (25 kDa), they had comparable or even higher gene transfection efficiency. Besides that, lignin graft polymers with interesting thermogelling properties have also been developed in our group, which have potential drug delivery and controlled release abilities [19]. However, there have been limited reports on cationic copolymers based on lignin for gene delivery [18,20].

This paper reports an in-depth study on lignin-PDMAEMA graft copolymers as nonviral gene delivery vector, to discover the relationship between the gene transfection performance of these copolymers and their structural factor, and to optimize such kind of system for different cell lines and conditions accordingly so as to prompt the development of lignin-based biomaterials.

## 2. Experimental Section

### 2.1. Materials

Lignin, alkali (Mn = 5000, Mw = 28,000) was purchased from Sigma-Aldrich (Saint Louis, MO, USA). Acetic anhydride (99.5%), pyridine (99.8%), 2-bromoisobutyryl bromide (98%), anhydrous *N*,*N*′-dimethylacetamide (99.8%, DMA) and *N*,*N*′-dimethylformamide (DMF, AR grade) were purchased from Sigma-Aldrich. The reagents and monomers for carrying out ATRP include 2-(dimethylamino)ethyl methacrylate (98%, DMAEMA), 1,1,4,7,10,10-hexamethyltriethylenetetramine (97%, HMTETA), CuBr (98%), 1.4-dioxane (AR grade) and tetrahydrofuran (AR grade, THF) were all purchased from Sigma-Aldrich. The deuterated solvents for NMR measurements like D_2_O and DCl solution 35 wt% in D_2_O (D, 99.5%), acetone-d_6_, and DMSO-d_6_ were supplied by Cambridge Isotope Laboratories (Tewksbury, MA, USA). The Luciferase kit was supplied by Promega (Madison, WI, USA). The reagent for cytotoxicity test, 3-(4,5-Dimethylthiazol-2-yl)-2,5-diphenyl tetrazolium bromide (MTT), was purchased from Sigma-Aldrich. Penicillin and streptomycin were from Sigma-Aldrich too.

### 2.2. Quantification of Total Hydroxyl Content in Lignin via Acetylation

To determine the total content of hydroxyl groups including aliphatic and phenolic ones, the dry lignin powder was treated with acetic anhydride in suitable organic media under mild conditions. Briefly, 400 mg of dried lignin (0.08 mmol) was dissolved in 16 mL of pyridine/acetic anhydride (1:1, *v*/*v*). The reaction solution was stirred for 48 h under nitrogen at room temperature. After that, the reaction solution was added dropwise with stirring to 300 mL of ice-water. The precipitated acetylated lignin was collected by centrifugation, washed three times with ice-water 100 mL, freeze-dried and further dried under vacuum at 50 °C. The ^1^H NMR measurement was then carried out. By comparing the signals (due to acetate ester formed between acetic anhydride and aliphatic/aromatic hydroxyl groups) of acetylated lignin with that of external reference (ethyl acetate), the content of hydroxyl groups in lignin was calculated to be 37.05 moles per mole of lignin (aliphatic -OH 59.8% and aromatic -OH 40.2%, respectively). The value was used to estimate the amount of required 2-bromoisobutyryl bromide for the modification of lignin.

### 2.3. Synthesis of Lignin-Based Macroinitiators

The synthesis procedures for the following two lignin-based macroinitiators are described as examples, which had a final modification degree (MD) of 3.0% and 100%, respectively.

#### 2.3.1. Synthesis of LnMI-a (MD 3.0%)

Lignin (5.0 g, 1.0 mmol, containing -OH 37.05 mmol) was weighed into a reaction flask, and dried at 100 °C under vacuum for one day. The dried lignin was cooled down to room temperature under a nitrogen atmosphere. After that, the lignin powder was fully dissolved by injecting anhydrous DMA (50 mL) into the flask under rapid stirring. Then 2-bromoisobutyryl bromide (5.679 g, 24.7 mmol, 3.05 mL) in 15 mL of anhydrous DMA was added dropwise into the lignin solution under rapid stirring over a period of 1 h. The reaction mixture was stirred for 2 days at room temperature under a N_2_ atmosphere. Thereafter, the reaction mixture was precipitated in 1 L of ether. The organic solution was poured out of the beaker, and the sticky tan gel-like solid left at the bottom of the beaker was re-dissolved in 30 mL of DMF and the solution was precipitated in 1 L of deionized water. The precipitate was collected by centrifugation, washed with 50 mL of deionized water twice and freeze-dried, giving the lignin macroinitiator as a brownish powder (3.94 g, yield: 45.4%). ^1^H NMR (DMSO-d_6_) of LnMI-a: δ (ppm) 1.4–2.2 (-CH_3_ of initiation group), 3.5–4.3 (CH_3_O-), 6.0–8.0 (aromatic protons of lignin).

#### 2.3.2. Synthesis of LnMI-h (MD 100.0%)

Lignin (5.0 g, 1 mmol, containing -OH 37.05 mmol) was weighed into a reaction flask, and dried at 100 °C under vacuum for one day. The dried lignin was cooled down to room temperature under a nitrogen atmosphere. After that, the lignin powder was fully dissolved by injecting anhydrous THF (160 mL) into the flask under rapid stirring. TEA (10.00 g, 98.8 mmol, 13.8 mL) was then added into the lignin solution. Subsequently, 2-bromoisobutyryl bromide (25.56 g, 111.2 mmol, 13.74 mL) in 20 mL of anhydrous THF was added dropwise into the lignin solution under rapid stirring over a period of 1 h. The reaction mixture was stirred for 2 days at room temperature under N_2_ atmosphere. Thereafter, the reaction mixture was precipitated in 1 L of ether. The organic solution was poured out of the beaker, and the sticky tan gel-like solid left at the bottom of the beaker was re-dissolved in 30 mL of DMF and the solution was then precipitated in 1 L of deionized water. The precipitate was collected by centrifugation, washed with 50 mL of deionized water twice and freeze-dried, giving the lignin macroinitiator as a brownish powder (8.92 g, yield: 84.8%). ^1^H NMR (DMSO-d_6_) of LnMI-h: δ (ppm) 1.4–2.2 (-CH_3_ of initiation group), 3.5–4.3 (CH_3_O-), 6.0–8.0 (aromatic protons of lignin).

### 2.4. Synthesis of Lignin-PDMAEMA Graft Copolymers

The synthesis procedures for LP-01 starting from LnMI-a (MD 3.0%) macroinitiator is described as an example.

Lignin-macroinitiator LnMI-a (MD = 3.0%) (251.3 mg, containing 0.06 mmol of -Br) and DMAEMA (0.943 g, 6 mmol, 1.011 mL) were added into a dry flask. The flask was sealed with a septum and subsequently purged with dry N_2_ for 5 min. Then, HMTETA (13.8 mg, 0.06 mmol, ca. 16 µL) in 3 mL of thoroughly degassed DMF was injected into the reaction flask with a degassed syringe. The mixture was stirred at room temperature and purged with dry N_2_ for 40 min. Thereafter, a suitable amount of catalyst CuBr (86.1 mg, 0.06 mmol) was added under a N_2_ atmosphere, and the reaction mixture was purged with dry N_2_ for another 10 min at room temperature. The mixture was then stirred for 48 h at 65 °C in an oil bath. The experiment was stopped by opening the flask and exposing the catalyst to air. The final tan mixture was diluted with THF and passed through a short neutral Al_2_O_3_ column with THF as eluent to remove copper catalyst. The resulting eluate solution was concentrated to about 10 mL and precipitated in 1 L of hexane. The precipitate was collected by centrifugation, washed with hexane, and dried under a vacuum at 40 °C, giving the final brownish product LP-01 (420 mg, yield 23%).

The ATRP procedures and reaction conditions for synthesis of other LPs are similar to those for LP-01. The main difference will be the modification degree of the lignin-based macroinitiator, the molar ratio of monomer/-Br and the reaction medium.

### 2.5. In Vitro Transfection and Luciferase Assay

Transfection studies were performed in COS7, MDA-MB-231 and HeLa cells using the plasmid pRL-CMV as reporter gene. In brief, 24-well plates were seeded with cells at a density of 5 × 10^4^/well 24 h before transfection. Samples (DNA complexes) at various N/P ratios were prepared by adding different samples into DNA solutions dropwise, followed by vortexing and incubation for 30 min at room temperature before transfection. At the time of transfection, the medium in each well was replaced with 300 µL reduced-serum medium or normal complete DMEM medium. The complexes were added into the transfection medium and incubated with cells for 4 h under standard incubator conditions. After 4 h, the medium was replaced with 500 µL of fresh medium supplemented with 10% FBS, and the cells were further incubated for an additional 20 h under the same conditions, resulting in a total transfection time of 24 h. Cells were washed with PBS twice, lysed in 100 L of cell culture lysis reagent (Promega). The luciferase activity in cell extracts was measured using a luciferase assay kit (Promega) on a single-well luminometer (Lumat LB 9507, Berthold Technologies, Bad Wildbad, Germany) for 10 s. The relative light units (RLU) were normalized against protein concentration in the cell extracts, which was measured using Commassie PlusTM Protein Assay Reagent (Pierce). Absorption was measured on a microplate reader (Infinite M200 PRO, TECAN, Switzerland) at 595 nm and compared to a standard curve calibrated with BSA. Results are expressed as relative light units per milligram of cell protein lysate (RLU/mg protein).

Other experimental methods such as proton nuclear magnetic resonance spectroscopy (^1^H NMR), elemental analysis (EA), dynamic light scattering (DLS) and zeta-potential measurements, plasmid preparation, gel retardation assay and cell viability assay etc. can be found in Supplementary Materials.

## 3. Results and Discussion

### 3.1. Preparation of ATRP-Initiator Modified Lignin with Various Modification Degree

Lignin is the only natural and renewable aromatic (phenolic) biopolymer produced mainly in the pulp and paper industries. It is heterogeneous, irregular, crosslinked and highly branched in composition and structure. Generally, lignin can be regarded as an extremely complex matrix composed of three basic phenylpropane units (primarily *p*-hydroxy phenyl (*H*), guaiacyl (*G*), and syringyl (*S*)), which are derived from three precursors (*p*-coumaryl, coniferyl and sinapyl alcohols, respectively), and are bonded together by a set of different linkages [21,22,23,24]. Though aromatic and relatively hydrophobic in nature, it contains a series of functional groups such as methoxyl, carbonyl and hydroxyl (alcohol and phenol) etc. By choosing suitable reactions to take place at these functional groups, this aromatic biopolymer can be modified in both physical and chemical properties, forming desired biomaterials [25,26].

**Figure 1:**
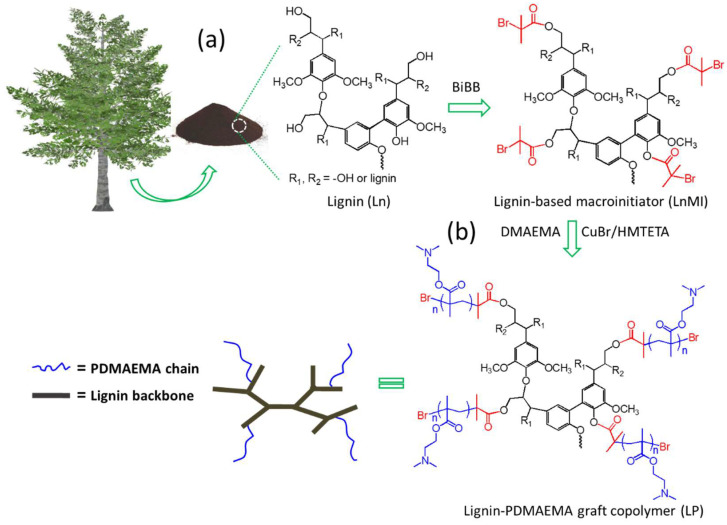
Synthetic procedures for (**a**) conversion of biomass lignin into lignin-based macroinitiator (LnMI) and (**b**) synthesis of lignin-based graft copolymer (LP) via Cu(I)-mediated ATRP of DMAEMA.

Similar to our reported method to obtain cyclodextrin copolymers through “grafting from” approach [27,28,29], herein lignin backbone was firstly grafted with ATRP initiator fragment, -OCO-C(CH_3_)_2_Br by reacting the aliphatic or phenolic hydroxyl functional groups (or both) on the lignin with 2-bromoisobutyryl bromide (BiBB) under anhydrous conditions to form lignin-based macroinitiators [18,19]. Since there is still a lack of fundamental understanding of the modification degree (MD, the percentage of lignin hydroxyl groups converted into ATRP initiation sites) of this kind of lignin-based macroinitiator for the properties and gene transfection performance of the resultant lignin graft copolymers after ATRP, it is necessary to synthesize a series of lignin-based macroinitiators with broad modification degrees for comprehensive study and comparison according to the pathway shown in Figure 1a.

Based on our previous results, the lignin in this work contains total hydroxyl group (-OH) of 37.05 moles per mole of lignin on average including aromatic -OH of 40.2% and aliphatic -OH of 59.8%, respectively (based on the average molecular weight of lignin being 5000 Da). By adjusting conditions like the molar ratio of BiBB/lignin, reaction medium and catalyst etc. the lignin macroinitiators with desired modification degree could be obtained. The selected synthesis conditions and characterization results for these macroinitiators with varied modification degrees ranging from 3.0 to 100% were summarized in Table 1 and Table 2.

**Figure 2:**
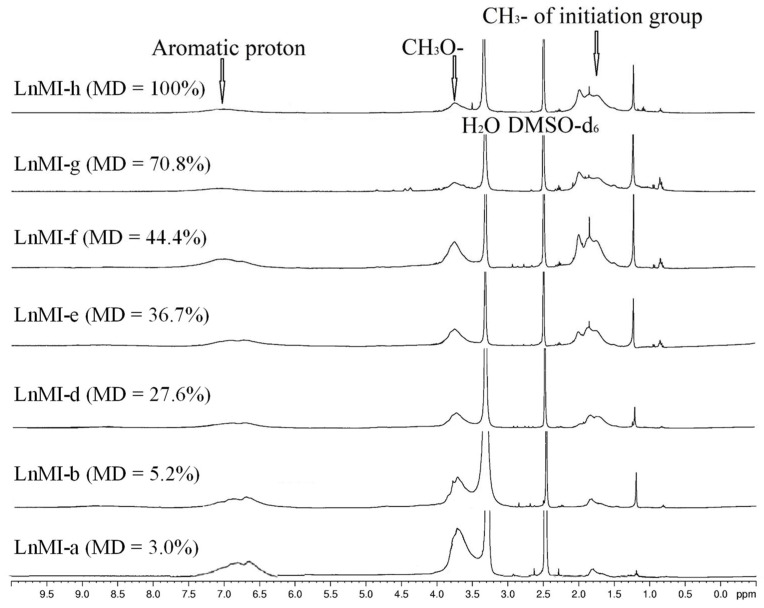
^1^H NMR characterization results for lignin-based macroinitiators (LnMIs) (DMSO-d_6_, 400 MHz, 25 °C). Data for LnMI-f and LnMI-h were reported previously [18]. The solvent residual peak (δ 2.5 ppm) and the water peak (δ 3.3 ppm) were labelled in these spectra.

The ^1^H NMR spectra for LnMIs with varied modification degrees from 3% to 100% were shown in Figure 2. Three kinds of proton signals can be clearly identified. The signal at 1.3–2.3 ppm is due to the proton of bromoisobutyryl ester group, -OCO-C(CH_3_)_2_Br, showing that the ATRP initiation site was successfully grafted onto the lignin backbone. The broad proton signal at 6.0–8.0 ppm and another signal at 3.5–4.2 ppm is due to the aromatic and methoxy (-CH_3_O) groups of lignin, respectively. If the integration of aromatic proton signal was set to be a fixed value, it can be observed that the integration of the proton signal due to the initiation site increased with increasing the MD. By comparing the integration of the proton signals due to initiation site to those of 100% modified macroinitiator (LnMI-h), the MD of LnMI could be calculated. Selected characterization results can be found in Table 2 where the MD, the number of initiation site (-OCO-C(CH_3_)_2_Br) per LnMI macromolecule and average Mn are key structural features of the lignin-based macroinitiators.

### 3.2. Preparation of Lignin-PDMAEMA Graft Copolymers with Varied Grafting Degree and Cationic Chain Length through ATRP

A series of lignin-PDMAEMA graft copolymers (LPs) were synthesized through ATRP of monomer DMAEMA in the presence of lignin-based macroinitiators under a nitrogen environment using CuBr /HMTETA as catalyst system according to our previously reported methods (Figure 1b) [18,19,27,28,29]. To carry out ATRP reactions in this study, the solubility of LnMIs should be considered for choosing a suitable solvent as reaction medium. The solubility of LnMIs depend on their modification degree (MDs). Generally, the LnMI with higher MD tends to be soluble in less polar solvent. For example, the initiator LnMI-a with a low MD of 3.0% dissolves well in DMF but has poor solubility in less polar THF and dioxane. Therefore, DMF was chosen as reaction medium for synthesis of copolymers LP-01 and LP-02. For the other initiators with MD > 3.0%, either dioxane or THF can be used as ATRP reaction media. In this paper, total 20 LPs were prepared. These copolymers possessed varied grafting degrees and cationic chain length determined by the lignin macroinitiator used for the synthesis and the ATPR conditions. Systematic studies on these copolymers could help us to make clear the influence of the structural parameters on copolymers’ properties when they were used as biomaterials. Note, in this work, the grafting degree (GD) of LP is equal to MD of the corresponding LnMI. The polymerization conditions and selected characterization results were summarized in Table 3 and Table 4, respectively.

**Figure 3:**
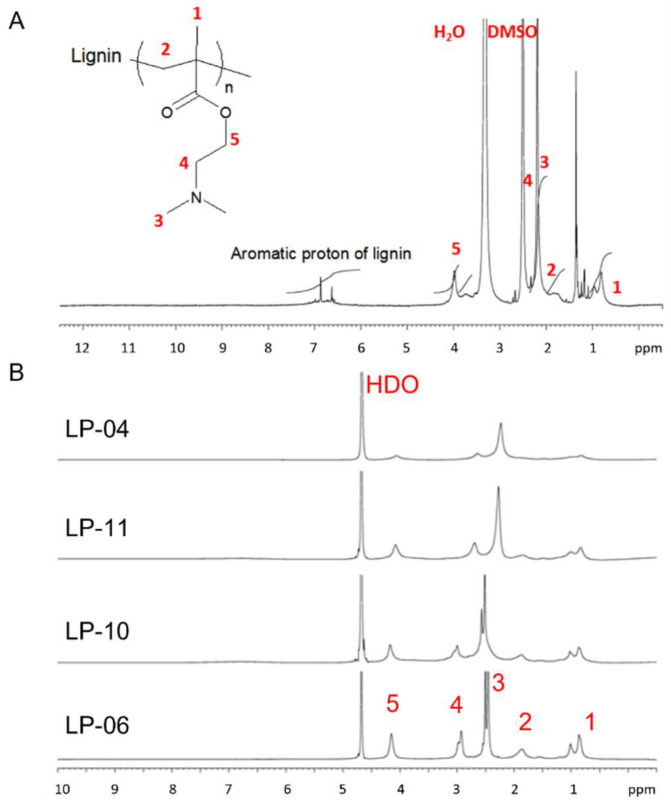
(**A**) ^1^H NMR characterization result for lignin-PDMAEMA graft copolymer LP-02 (DMSO-d_6_, 400 MHz, 25 °C). (**B**) ^1^H NMR characterization results for lignin-PDMAEMA graft copolymers LP-04, LP-06, LP-10, and LP-11 in D_2_O containing DCl (0.05 mol/L), respectively (400 MHz, 25 °C). For (**A**), the solvent residual peak (δ 2.5 ppm) and the water peak (δ 3.3 ppm) were labelled. For (**B**), the solvent residual peak was labelled at δ 4.7 ppm.

After grafting PDMAEMA arms onto lignin backbone through the “grafting from” approach described above, the biomass lignin becomes processable. The obtained lignin-PDMAEMA copolymer was found to have good solubility in common organic solvent such as ethanol, acetone, THF, chloroform and 1,4-dioxane, etc. A typical ^1^H NMR spectrum for copolymer LP-02 (obtained from initiator LnMI-a, under [M]_0_/[I]_0_/[Cu]_0_/[L]_0_ = 200/1/1/1) in DMSO-d_6_ was shown in Figure 3A as an example. The signals due to aromatic protons of lignin backbone (δ 6–8 ppm) and the protons of DMAEMA unit (δ 4.1, 2.5, 2.3 and 0.5–2.0 ppm) were clearly observed. Among these signals, the peak at 2.5 ppm due to DMAEMA unit was overlapped with the residual solvent peak of DMSO-d_6_. Most of the LPs also dissolved well in DI water and PBS buffer solution (1×). However, the water solubility of the graft copolymer depended on its composition. Generally, the copolymers with more grafted DMAEMA units exhibited better water solubility. Further study showed that when the copolymers were treated with dilute HCl aqueous solution (0.1 M), its water solubility was improved. For example, the copolymer LP-05 (obtained from initiator LnMI-d, under [M]_0_/[I]_0_/[Cu]_0_/[L]_0_ = 30/1/1/1) did not dissolve in water as well as other copolymers. However, it became very soluble in dilute HCl aqueous solution (0.1 M). Therefore, in the case that the LP copolymer has low solubility in the water, it is suggested to add several drops of dilute HCl aqueous solution (0.1 M) to the mixture until the copolymer fully dissolves.

^1^H NMR results of lignin-PDMAEMA graft copolymers in D_2_O containing DCl (0.05 mol/L) were shown in Figure 3B. The peaks attributed to the -CH_3_ and -CH_2_ adjacent to N (peaks 3 and 4) were chemically shifted with the change of the DMAEMA content in grafted polymers. Both peaks moved towards higher field with the increase of the DMAEMA content in copolymer. This might be attributed to the π electron of the aromatic rings of lignin which showed an electronic shielding effect on the N_3_^+^ moiety (referring to the protonated tertiary amine of DMAEMA unit). The electron distribution shifted from aromatic rings of lignin to N_3_^+^ moiety of DMAEMA unit, causing the electron density of -CH_3_ and -CH_2_ to increase. In this study, the average DMAEMA repeating units grafted onto lignin macromolecule for copolymers LP-10 and LP-04 are 45.4 and 80.6, respectively (according to Table 2 and Table 4). The content of DMAEMA repeating unit in copolymer was also well reflected by the N% in copolymer as seen in Table 4. For LP-10, the peak 4 and peak 3 are located around δ 3.01 and 2.52 ppm, respectively. By comparison, for LP-04, both peaks are shifted to δ 2.66 (peak 4) and 2.25 (peak 3) ppm, respectively. When the content of DMAEMA units further increased, the π electron on the aromatic rings of lignin became insufficient to affect the N_3_^+^ moiety. Therefore, the peaks 3 and 4 will move towards lower field. Such trend was observed in the case of copolymers LP-11 and LP-06. In these copolymers, the average DMAEMA repeating units per lignin macromolecule are 135.3 and 232.6, respectively. For LP-11, the peak 4 and peak 3 are located around δ 2.68 and 2.26 ppm, respectively. While, for LP-06, both peaks are down-filed shifted to δ 2.92 (peak 4) and 2.49 (peak 3) ppm, respectively.

### 3.3. pDNA Condensation Capability of Lignin Graft Copolymers

**Figure 4:**
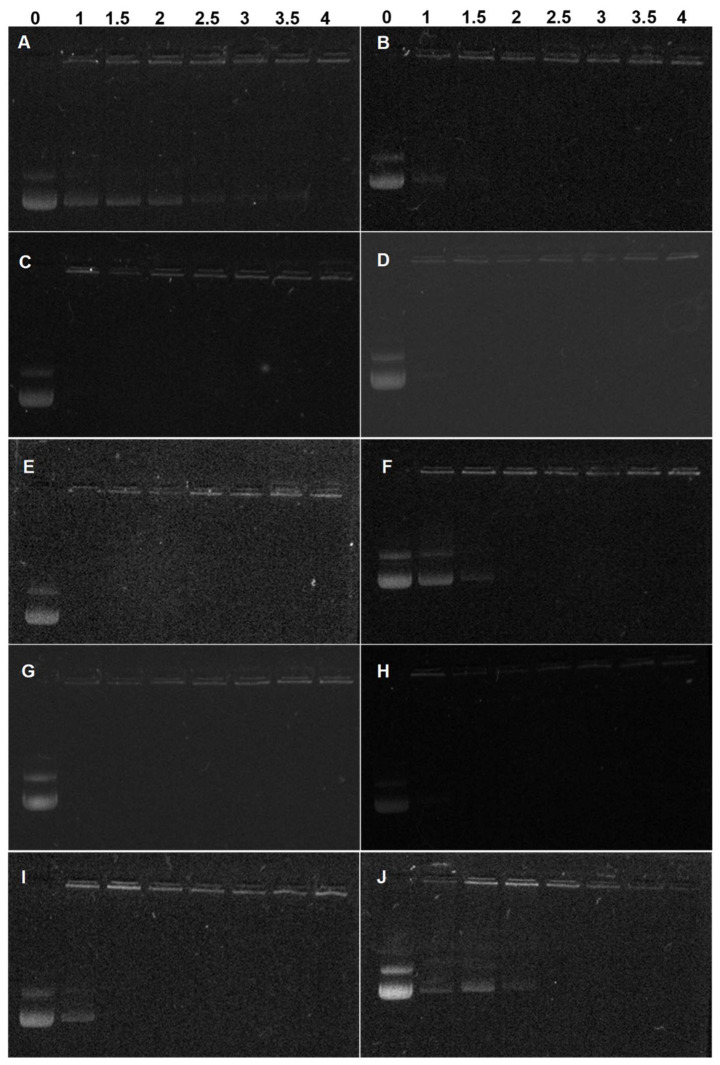
Electrophoretic mobility of pDNA in the polyplexes formed with lignin graft copolymers (**A**) LP-03, (**B**) LP-05, (**C**) LP-06, (**D**) LP-11, (**E**) LP-12, (**F**) LP-14, (**G**) LP-16, (**H**) LP-17, (**I**) LP-20 and (**J**) PEI (25 kDa) at various N/P ratios; Data for (**F**) LP-14 and (**G**) LP-16 were reported previously [18].

**Figure 5:**
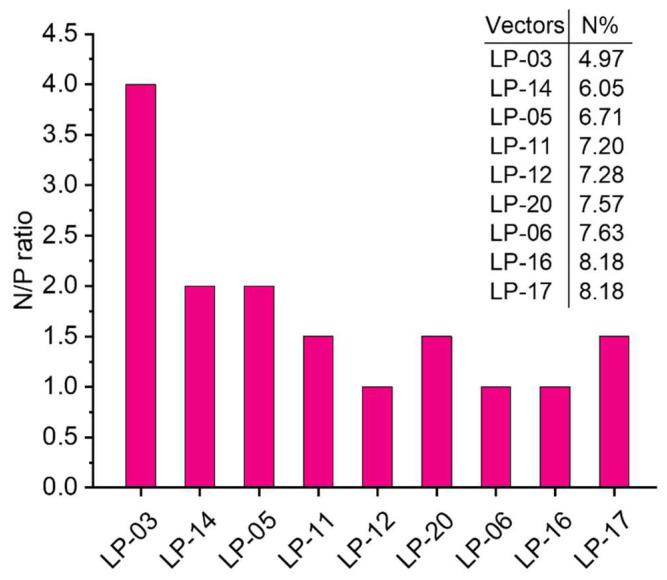
Relation between N/P ratio at which complete DNA retardation was observed and the nitrogen content (N%) of lignin-PDMAEMA graft polymer. Data for LP-14 and LP-16 were reported previously [18].

The DNA condensation induced by a cationic polymer is a prerequisite for successful gene delivery [30]. In graft copolymer of lignin-PDMAEMA, the DNA condensation ability is mainly dependent on the grafted cationic PDMAEMA arms because the lignin itself has no DNA condensation ability. Through the “grafting from” technology, the DNA condensation ability due to PDMAEMA arms and biocompatibility due to lignin was well combined in the resultant graft copolymers. Herein, the pDNA condensation capabilities of these lignin copolymers were evaluated by agarose gel electrophoresis, particle size and zeta potential measurements. Polyplexes were prepared by electrostatic interaction between the positively charged lignin-PDMAEMA copolymers and negatively charged pDNA at various N/P ratios. Figure 4A–I showed the gel retardation results for nine lignin-PDMAEMA copolymers with varied N content (N%). Branched PEI (25 kDa) was used as standard for comparison (see Figure 4J). It can be seen that different copolymer showed different N/P ratios at which complete DNA retardation could be observed, showing their pDNA condensation capabilities were different. Generally, the condensation capability of lignin-PDMAEMA copolymer was dependent on its N content. The higher the N%, the better condensation ability could be achieved. For example, LP-03 (N content = 4.97%) can fully retard the pDNA movement at N/P ratio of 4.0 (Figure 4A), while in the case of LP-17 (N content = 8.18%), N/P ratio of 1.5 was enough for complete DNA retardation (Figure 4I). This value was comparable to that of standard PEI 25 KDa with N/P ratio of 2.0 for complete DNA retardation (Figure 4J). This result showed the graft copolymer LP-17 has similar pDNA condensation capability to PEI 25K under the same experimental conditions. However, our further studied revealed that the DNA condensation ability of these lignin-PDMAEMA graft copolymers was not determined by the N content only. In order to investigate this interesting phenomenon, the variation of the copolymer’s DNA condensation ability with the N% was shown in Figure 5. It can be seen in Figure 5, though the copolymers were arranged in their N% (from lower to higher) for the X axis, that the N/P ratio for the complete DNA retardation (reflecting the DNA condensation ability) for the Y axis did not decrease continuously with increasing the N% ranging from 4.97 to 8.18. For example, LP-12 (N% = 7.28) showed better DNA condensation capability (N/P = 1.0) than LP-20 (N% = 7.57) with the N/P ratio of 1.5. This implied that besides the N content of the lignin-PDMAEMA graft copolymer, the structural factors might take effect on the copolymer’s pDNA condensation ability also.

Noteworthily, the lignin-PDMAEMA graft copolymers developed in this work possessed highly branched structure. They had structural differences in grafting degrees (GD, equal to modification degree of lignin macroinitiator, which determined the cationic PDMAEMA arm number per lignin macromolecule) and average degree of polymerization (DP) which determined the cationic PDMAEMA arm length since they were synthesized by using different lignin macroinitiator and under different ATRP conditions (Please refer to Table 3 and Table 4). These structural factors could affect the copolymer’s pDNA condensation capability. The copolymers LP-03, 05 and 06 which were synthesized from lignin initiator LnMI-d were examined. All of them had the same GD (which was corresponded to ca. 10.2 PDMAEMA arms per lignin macromolecule), but different DPs of 4.7, 11.6 and 22.8, respectively. Correspondingly, the N content for them was 4.97, 6.71 and 7.63%, respectively. It can be seen from Figure 5, these copolymers could fully retard the pDNA movement at N/P ratio of 4, 2 and 1, respectively. This observation showed that for these copolymers obtained from same lignin initiator, the pDNA condensation ability increased with the increase of cationic arm length. In this case, the N% of the graft copolymer was proportional to the cationic arm length. Another pair of copolymers obtained from same macroinitiator LnMI-f were LP-14 (DP = 5.5) and LP-16 (DP = 30.1). They showed N/P ratio of 2.0 and 1.0, respectively. This result was consistent with that observed for copolymers LP-03, 05 and 06.

When the copolymers obtained from different initiators were compared, the GD and DP together with the N% should be considered. The copolymer LP-12 (N% = 7.28) which showed a N/P ratio of 1.0 possessed a GD of 36.7% and a DP of 13.6. While the copolymer LP-20 (N% = 7.57) which showed a N/P ratio of 1.5 possessed a GD of 20.2% and a DP of 28.6. The copolymer LP-12 with shorter grafted arm but higher number of arms per lignin showed better pDNA condensation ability than copolymer LP-20 with longer grafted arm but lower number of arms per lignin, though the former had lower N% than the latter. In the case when the copolymers had very similar N%, the effect of structural difference on the pDNA condensation ability of the copolymer became easier to identify. For example, both LP-16 and 17 had quite similar N% of 8.18, however, the former (GD = 44.4%, DP = 30.1) condensed pDNA better than the latter (GD = 70.8%, DP = 21.1). The N/P ratio was 1.0 and 1.5 for LP-16 and 17, respectively.

**Figure 6:**
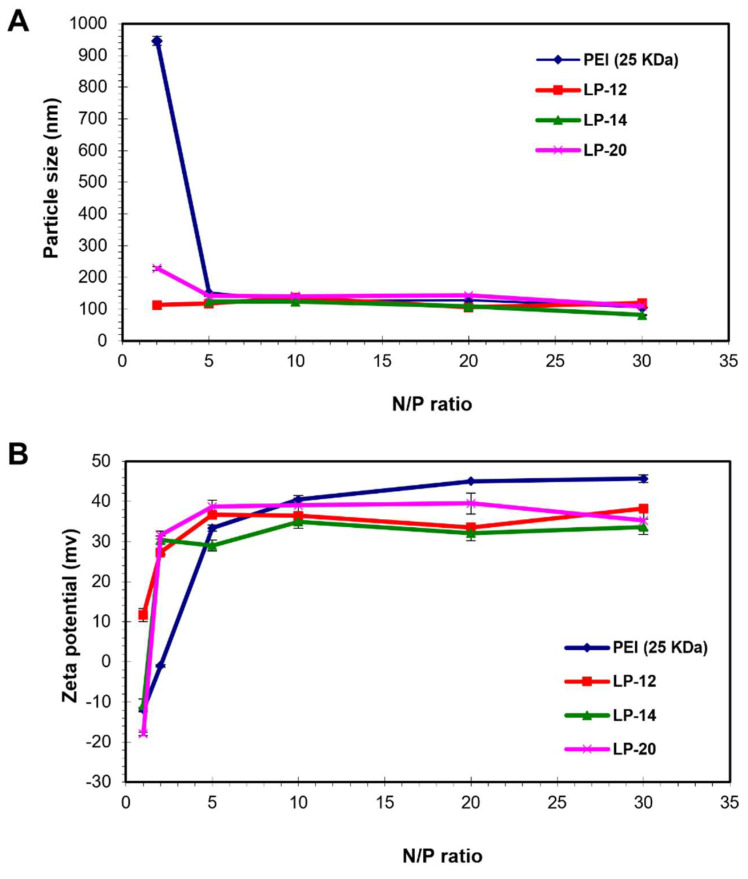
Particle size (**A**) and zeta potential (**B**) of the polyplexes formed between cationic lignin-PDMAEMA carriers (LP-12, LP-14 and LP-20) and pDNA in comparison with PEI/pDNA polyplexes at various N/P ratios. Data represent mean ± standard deviation (*n* = 2 for A, *n* = 3 for B). Data for LP-14 was reported previously [18].

All the lignin-PDMAEMA grafted polymers developed in this work could condense the pDNA into small nanoparticles. The data of particle size and zeta potential for three grafted polymers LP-12, 14 and 20 were shown in Figure 6 as examples. PEI 25K was also tested under the same conditions for comparison. With the increase of N/P ratio, the particle size of the resultant polyplexes decreased (Figure 6A). At the N/P ratio ranging from 5 to 30, the particle size of the polyplexes was relatively stable (around 70 to 150 nm dependent on the copolymer used for forming the complexes with pDNA). Generally, there was no obvious difference in particle size between the polyplexes formed with lignin-PDMAEMAs and PEI 25K. With the increase of N/P ratio, the zeta potential of these complexes increased. The zeta potential became relatively stable (around +30 to +40 mv dependent on the copolymer used for forming the complexes with pDNA) at the N/P ratio ranging from 10 to 30 (Figure 6B). In this range, the zeta potential of the polyplexes with lignin-PDMAEMAs was obviously lower than that of the polyplexes with PEI 25K.

### 3.4. Cytotoxic Effect of Lignin-PDMAEMA Copolymers on MDA-MB-231 and COS7 Cells

**Figure 7:**
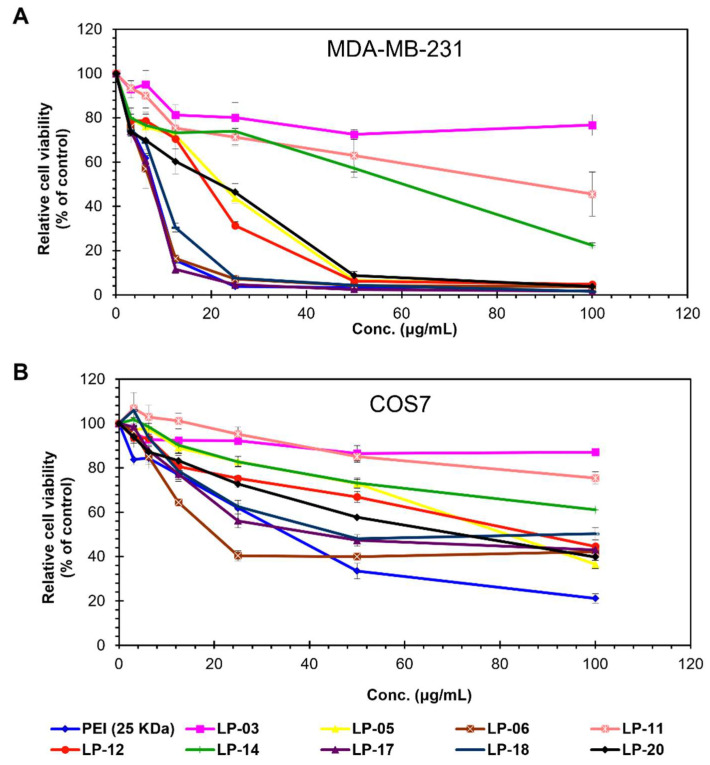
Cell viability assay of lignin-PDMAEMA copolymers in MDA-MB-231 (**A**) and COS7 (**B**) cells in comparison with branched PEI (25 kDa). Data represent mean ± standard deviation (*n* = 6). Data for LP-14 was reported previously [18].

Figure 7 showed the in vitro cytotoxic effect of lignin-PDMAEMA copolymers on MDA-MB-231 (A) and COS7 (B) cell lines in comparison with branched PEI (25 kDa). Generally, the cytotoxicity of lignin-PDMAEMA copolymers increased (reflected by the decrease in the relative cell viability) with the increase of copolymer’s concentration in the range of 0–100 µg/mL in both MDA-MB-231 and COS7 cell lines.

As can be seen in Figure 7, under the same copolymer concentration the cytotoxicity of lignin-PDMAEMA copolymers generally increased with the increase of copolymers’ N content in both MDA-MB-231 and COS7 cell lines. For example, LP-03 (N = 4.97%) showed the lowest toxicity among all the selected copolymers, while the LP-18 (N = 8.34%) gave a much higher cytotoxicity than LP-03. The cytotoxicity of LP-18 was comparable to branched PEI 25K under same condition in this study. The estimated IC_50_ values for LP-12, 14 and 20 in MDA-MB-231 cell line were 18, 68 and 24 μg/mL, respectively, while in COS7 cell line, the IC50 values were 96, 126 and 84 μg/mL for LP-12, 14 and 20, respectively. This could be attributed to the difference in tolerance of the two cell lines to toxic material. In MDA-MB-231 cell line, the relative cell viability was very sensitive to the concentration of lignin copolymer. It decreased dramatically when the copolymer concentration increased from 25 μg/mL to 50 μg/mL, as shown in Figure 7A. In COS7 cell line, the change of the relative cell viability with the copolymer concentration was obviously slower as compared to that in MDA-MB-231 cell line (Figure 7B). Generally, the cytotoxicity of the lignin-PDMAEMA copolymers was lower than that of PEI standard in both cell lines.

**Figure 8:**
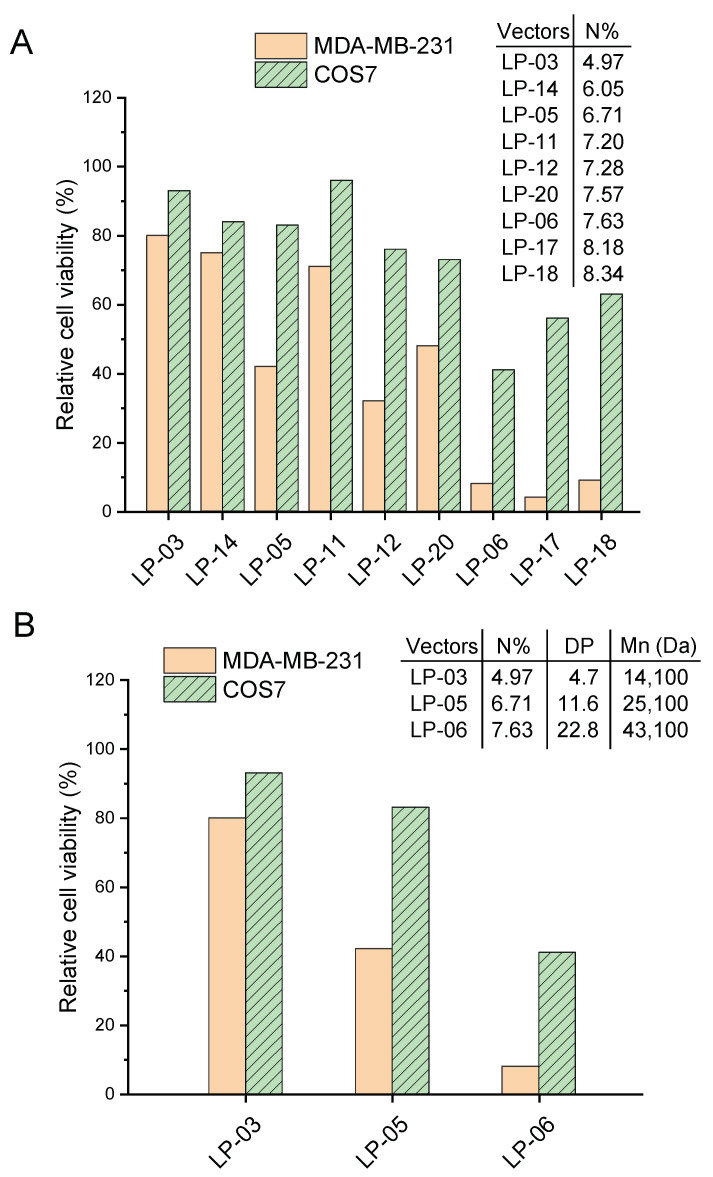
(**A**) Variation of the relative cell viability with the N content of the lignin-PDMAEMA copolymers in MDA-MB-231 and COS7 cell lines at the copolymer concentration of 25 μg/mL. (**B**) Variations of the relative cell viability with the degree of polymerization (DP) and molecular weight (Mn) for the selected lignin-PDMAEMA copolymers LP-03, 05 and 06 in MDA-MB-231 and COS7 cell lines at the copolymer concentration of 25 μg/mL. Data for LP-14 was reported previously [18].

To further analyze the relationship between the cytotoxicity of lignin-PDMAEMA copolymers and their N content, all the copolymers under study were arranged in their N% from lower to higher. The order in the format of LP (N%) was as follows: LP-03 (4.97), LP-14 (6.05), LP-05 (6.71), LP-11 (7.20), LP-12 (7.28), LP-20 (7.57), LP-06 (7.63), LP-17 (8.18) and LP-18 (8.34). Some representative copolymers were analyzed. Figure 8A showed the variation of the relative cell viability with the N content of these lignin-PDMAEMA copolymers in MDA-MB-231 and COS7 cell lines at a fixed copolymer concentration of 25 μg/mL. As can be clearly seen in the graph, some copolymers did not follow the general trend (i.e., the higher the N% at fixed copolymer concentration, the lower the relative cell viability) such as LP-11, 20, 17 and 18, in comparison with their neighboring copolymers. This phenomenon indicated that the copolymer’s structural factors obviously affected their cytotoxicity. When the copolymers obtained from different initiators were compared in cytotoxicity, both the difference in structure (cationic arm number and arm length) and N% should be considered. This phenomenon was similar to that for the lignin copolymer’s pDNA condensation ability as we observed and analyzed in previous section (see Figure 4 and Figure 5).

However, for copolymers obtained from same lignin initiator, their cytotoxicity was usually determined by PDMAEMA arm length only. In such cases, both the N% and molecular weight of copolymer were proportional to the PDMAEMA arm length. The copolymers LP-03, 05 and 06 which were synthesized from lignin initiator LnMI-d were highlighted here as an example. The variations of the relative cell viability with the degree of polymerization (DP) and molecular weight (Mn) of these lignin-PDMAEMA copolymers in MDA-MB-231 and COS7 cell lines at the fixed copolymer concentration of 25 μg/mL were shown in Figure 8B. With the increase of DP, the relative cell viability was decreased in both MDA-MB-231 and COS7 cell lines. This trend was consistent with expectation.

### 3.5. In Vitro Transfection Efficiency of Lignin-PDMAEMA Copolymers

**Figure 9:**
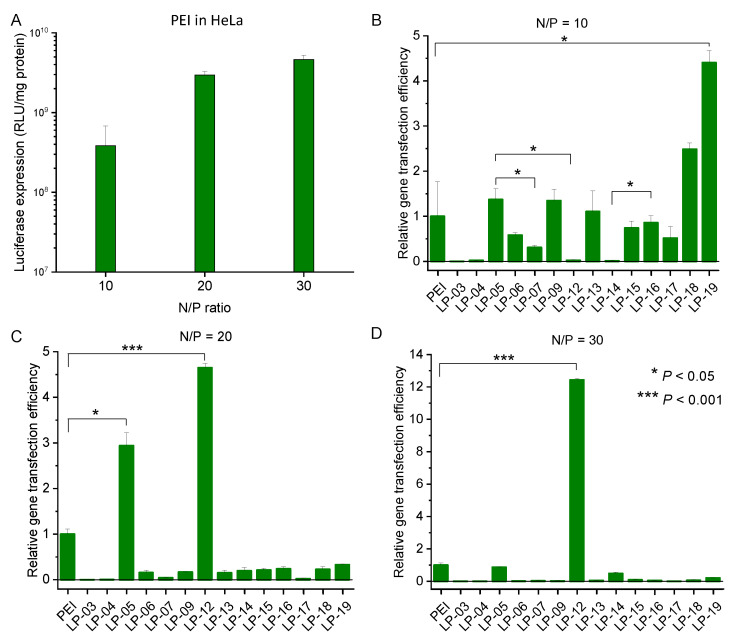
In vitro gene transfection efficiency of the complexes of PEI/pDNA in HeLa cells in the presence of serum at different N/P ratios (**A**). Relative gene transfection efficiency of the complexes of LP/pDNA in HeLa cell line in the presence of serum at N/P of 10 (**B**), 20 (**C**) and 30 (**D**). Data represent mean ± standard deviation (* *p* < 0.05, *** *p* < 0.001, *n* = 2).

In vitro gene transfection and luciferase assay was carried for lignin-PDMAEMA copolymers in three cell lines of HeLa, COS7 and MB-231 in the presence of serum. The copolymers included for this study were LP-03, 04, 05, 06, 07, 09, 12, 13, 14, 15, 16, 17, 18 and 19, which varied in N content in a broad range from 4.97 to 8.34% and possessed different PDMAEMA arm numbers and lengths (see Table 3 and Table 4). For an easy comparison of the gen transfection performance between these copolymers, the relative gene transfection efficiency for each copolymer was calculated, which was the ratio of luciferase expression between LP/pDNA and PEI 25K/pDNA under the same condition. In the cell line of HeLa (Figure 9), the copolymers from LP-03 to 07 were examined first. They were obtained from the same lignin macroinitiator LnMI-d (MD = 27.6%) and possessed the same number of cationic PDMAEMA arms per lignin but varied in arm length. The DP of these copolymers increased from LP-03 to 07. At N/P of 10 (Figure 9B), the gene transfection ability of this group of copolymers increased with the increase of DP. This should be due to the increasing pDNA condensation ability with DP. LP-05 showed the best gene transfection efficiency among these copolymers, which was comparable to that of PEI 25K standard. With a further increase in DP from LP-05 to 07, the copolymer’s gene transfection efficiency significantly decreased. This should be due to the increasing cytotoxicity of the copolymer when the cationic arm became longer. LP-05 was also compared with the analogues obtained from different lignin macroinitiator. At the N/P of 10, LP-05 (N% = 6.71, GD = 27.6%, DP = 11.6) showed obviously higher gene transfection efficiency than LP-12 (N% = 7.28, GD = 36.7%, DP = 13.6) and LP-14 (N% = 6.05, GD = 44.4%, DP = 5.5). This should be due to the complexity of the in vitro gene transfection process which was affected by a combination of factors such as the copolymer’s properties, polyplexes properties and the interactions between polyplexes and cells etc. Similar to the phenomena found for the copolymer’s pDNA condensation ability and cytotoxicity to different cell lines, the copolymer’s gene transfection efficiency should be affected by both the copolymer’s structural factors and N content. At N/P ratio of 20 (Figure 9C), LP-05 still performed well with the gene transfection efficiency ca. three times as high as PEI standard. The copolymer LP-12′s gene transfection efficiency increased significantly, which was near five times as high as PEI standard. Other copolymers showed obviously lower gene transfection performance as compared with PEI (the relative gene transfection efficiency is close to 0). At N/P ratio of 30 (Figure 9D), the copolymer LP-12 showed a gene transfection efficiency more than 11 times higher than PEI. While for the rest copolymers, only LP-05 and 14 showed a gene transfection efficiency comparable to that of PEI standard (ca. 0.9 and 0.5 times of PEI’s efficiency, respectively).

The copolymers LP-14, 15 and 16 also showed an interesting trend in gene transfection efficiency. They were synthesized from LnMI-f (MD = 44.4%) and possessed varied DP of 5.5, 19.6 and 30.1, respectively. At lower N/P ratios of 10 and 20, the gene transfection increased with DP, which showed that in that case, the copolymers with longer arms performed better at lower N/P ratios. However, at higher N/P ratio of 30, LP-14 showed the highest gene transfection efficiency, which indicated that in that case, the copolymers with shorter arms performed better at higher N/P ratios.

**Figure 10:**
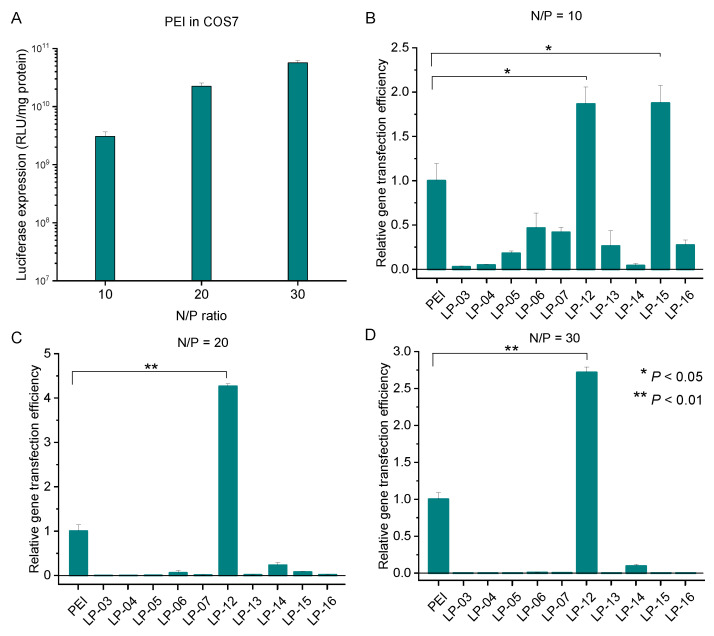
In vitro gene transfection efficiency of the complexes of PEI/pDNA in COS7 cells in the presence of serum at different N/P ratios (**A**). Relative gene transfection efficiency of the complexes of LP/pDNA in COS7 cell line in the presence of serum at N/P of 10 (**B**), 20 (**C**) and 30 (**D**). Data represent mean ± standard deviation (* *p* < 0.05, ** *p* < 0.01, *n* = 2).

In the cell line of COS 7 (Figure 10), the copolymer LP-12 exhibited the best gene transfection capability among all the copolymers, which was ca. two to four times as high as that of standard PEI at the N/P ratios from 10 to 30.

**Figure 11:**
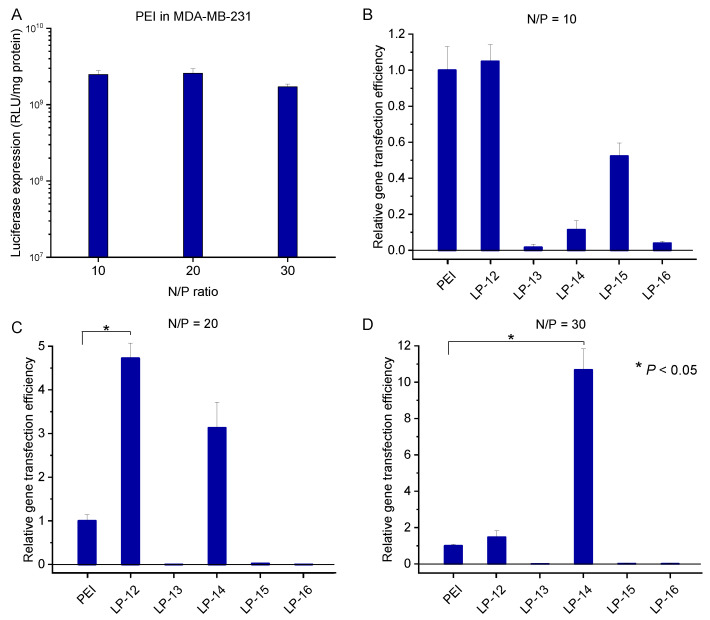
In vitro gene transfection efficiency of the complexes of PEI/pDNA in MDA-MB-231 cells in the presence of serum at different N/P ratios (**A**). Relative gene transfection efficiency of the complexes of LP/pDNA in MDA-MB-231 cell line in the presence of serum at N/P of 10 (**B**), 20 (**C**) and 30 (**D**). Data represent mean ± standard deviation (* *p* < 0.05, *n* = 2).

In the cell line of MDA-MB-231 (Figure 11), at the lower N/P ratios of 10 and 20, LP-12 was the best copolymer in gene transfection among all the copolymers. Its gene transfection efficiency was comparable to PEI at N/P of 10. The value was further increased to be nearly five times higher than PEI at N/P of 20. When N/P ratio was further increased to 30, its gene transfection efficiency decreased to ca. 1.7 times of PEI. By comparison, at the N/P ratio of 30, LP-14 showed the highest gene transfection efficiency, which was ca. 11 times higher than PEI. From the evaluation of these lignin-PDMAEMA copolymers in the three different cell lines, it can be found that both LP-12 and 14 are most promising nonviral gene transfection vectors.

## 4. Conclusions

In summary, the novel lignin-based graft copolymer LP was comprehensively studied for use as nonviral gene delivery vector. A series of lignin-based macroinitiators (LnMI-a to h) with different modification degrees (MDs) were synthesized (MD from 3.0 to 100%) in an easy and efficient way. These macroinitiators can successfully initiate atom transfer radical polymerization (ATRP) of monomer DMAEMA to form lignin-PDMAEMA graft copolymers through the “grafting from” approach under varied reaction conditions. As a result, a series of LPs with different grafting degrees (equal to modification degree of LnMI), cationic PDMAEMA arm length, molecular weight, and nitrogen content (N%) were obtained. It was confirmed that both the structural and composition factors exerted influence on the copolymers′ gene transfection efficiency. Generally, a copolymer with shorter PDMAEMA arm length and higher grafting degree demonstrated better gene transfection performance. In this study, copolymer LP-12 (N content of 7.28% and DP of 13.6) synthesized from lignin initiator LnMI-e (MD 36.7%) was found to perform well in all the cell lines of COS7, HeLa and MDA-MB-231. Its gene transfection efficiency was generally several times higher than that of the standard branched PEI 25K at N/P ratios from 10 to 30. However, in the cell line MDA-MB-231 at a higher N/P ratio of 30, the copolymer LP-14 (N content of 6.05% and DP of 5.5) synthesized from lignin initiator LnMI-f (MD 44.4%) showed best gene transfection efficiency among all the copolymers. Its gene transfection efficiency was ca. 11 times higher than that of the standard branched PEI 25K. This work demonstrated that, although the content of N element (N%), which is due to the grafted PDMAEMA chains, primarily determines the gene transfection efficiency of the LPs, it is not the only factor in explaining the performance of such copolymers with a branched structure. Structural factors of these copolymers such as grafting degree and cationic chain length could have a profound effect on the copolymer performance on gene transfection efficiency. Through carefully adjusting these factors, the gene transfection efficiency of the LPs could be modulated and optimized for different cell lines, which could make this new type of biomass-based biomaterial an attractive choice for various gene delivery applications.

## Figures and Tables

**Table 1:** The selected conditions for synthesis of lignin-based macroinitiators (LnMIs) with different modification degrees ^1^.

Lignin-Based Macroinitiators ^2^	The Initial Molar Ratios of BiBB/Lignin	Reaction Medium ^3^	TEA (mmol) ^4^	MD (%) ^5^
LnMI-a	24.7	DMA	\	3.0
LnMI-b	34.6	DMA	\	5.2
LnMI-c	36.2	DMA	\	20.2
LnMI-d	49.4	DMA	\	27.6
LnMI-e	64.8	DMA	\	36.7
LnMI-f	114.5	DMA	\	44.4
LnMI-g	229.0	DMA	\	70.8
LnMI-h	111.2	THF	98.8	100

^1^ The amount of dried lignin for modification was fixed at 5.0 g (1 mmol, based on the average molecular weight of lignin being 5000 Da). The reaction was carried out at room temperature for 2 days under N_2_ atmosphere. ^2^ Data for LnMI-f and LnMI-h were reported previously [18]. ^3^ Anhydrous reagents were used. ^4^ Anhydrous TEA was used as catalyst for the esterification reaction. ^5^ Modification degree (MD) is defined as the percentage of lignin hydroxyl groups converted into ATRP initiation sites. MD was calculated according to ^1^H NMR by comparing the integration of the proton signals due to initiation site to those of 100% modified macroinitiator, LnMI-h.

**Table 2:** The selected characterization results for lignin-based macroinitiators (LnMIs) with different modification degrees.

Lignin-Based Macroinitiators ^1^	MD (%) ^2^	Number of -Br per LnMI ^3^	Mn (Da) ^4^	EA Results (%)			Yield (%)
				C	N	H	
LnMI-a	3.0	1.1	5166	56.09	0.73	5.02	45.4
LnMI-b	5.2	1.9	5286	63.03	0.28	5.48	46.1
LnMI-c	20.2	7.5	6115	57.69	0.59	5.16	50.1
LnMI-d	27.6	10.2	6523	55.30	0.40	4.40	47.8
LnMI-e	36.7	13.6	7025	54.89	0.49	4.96	53.4
LnMI-f	44.4	16.5	7451	53.46	0.51	4.83	63.8
LnMI-g	70.8	26.2	8908	52.04	0.94	4.96	63.5
LnMI-h	100	37.1	10,519	49.91	0.85	4.75	84.8

^1^ Data for LnMI-f and LnMI-h were reported previously [18]. ^2^ Calculated according to ^1^H NMR by comparing the integration of the proton signals due to initiation site to those of 100% modified macroinitiator, LnMI-h. ^3^ Calculated according to MD and the total hydroxyl amount (37.05 mmol) per mmol of starting lignin. It means the number of initiation site, -OCO-C(CH_3_)_2_Br, per LnMI macromolecule on average after modification. ^4^ Calculated according to MD and the average Mn of starting lignin (5000 Da).

**Table 3:** The selected polymerization conditions for lignin-PDMAEMA copolymers ^1^.

Lignin PDMAEMA	Initiator	Initial [M]_0_/[I]_0_/[Cu]_0_/[L]_0_ ^4^	Monomer (mmol)	Reaction Medium	Yield (%)
LP-01	LnMI-a (MD = 3.0%)	100:1:1:1	5.0	DMF/3 mL	23
LP-02		200:1:1:1	10.0	DMF/3 mL	17
LP-03	LnMI-d (MD = 27.6%)	10:1:1:1	1.3	THF/5 mL	42
LP-04		20:1:1:1	2.6	THF/5 mL	38
LP-05		30:1:1:1	3.9	THF/5 mL	42
LP-06		50:1:1:1	6.5	THF/5 mL	46
LP-07		100:1:1:1	13.0	THF/5 mL	26
LP-08 ^2^		100:1:1:1	13.0	Dioxane/3 mL	/
LP-09 ^2^		100:1:1:1	13.0	Dioxane/4 mL	57
LP-10	LnMI-b (MD = 5.2%)	50:1:1:1	1.45	Dioxane/2 mL	36
LP-11		100:1:1:1	2.9	Dioxane/2 mL	56
LP-12	LnMI-e (MD = 36.7%)	50:1:1:1	6.5	Dioxane/4 mL	38
LP-13		100:1:1:1	13.0	Dioxane/4 mL	59
LP-14 ^3^	LnMI-f (MD = 44.4%)	50:1:1:1	6.5	Dioxane/4 mL	21
LP-15 ^3^		100:1:1:1	13.0	THF/4 mL	28
LP-16 ^3^		100:1:1:1	13.0	Dioxane/4 mL	44
LP-17	LnMI-g (MD = 70.8%)	100:1:1:1	13.0	Dioxane/4 mL	38
LP-18	LnMI-h (MD = 100%)	50:1:1:1	6.5	Dioxane/4 mL	43
LP-19 ^2^		100:1:1:1	18.0	Dioxane/3 mL	49
LP-20	LnMI-c (MD = 20.2%)	70:1:1:1	18.0	Dioxane/3 mL	55

^1^ The ATRP reaction was carried out at 65 °C for 2 days under N_2_ atmosphere. However, for LP-08 and 19, the ATRP reaction was carried out for 2 h and 1 h, respectively instead. ^2^ LP-08, 09 and 19 were treated with dilute HCl aqueous solution (0.1 M) to further improve their water solubility. ^3^ Data for LP-14,15 and 16 were reported previously [18]. ^4^ [M]_0_, [I]_0_, [Cu]_0_ and [L]_0_ represent the initial molar concentrations of monomer, total initiation sites, CuBr and HMTETA, respectively.

**Table 4:** The selected EA results for lignin-PDMAEMA copolymers.

Lignin-PDMAEMA	EA Results (%)			Mn (Da) ^1^	Number of DMAEMA Unit Per Arm ^1^
	N	C	H		
LP-01	3.93	55.45	6.57	8300	18.2
LP-02	6.81	59.13	8.54	20,500	88.9
LP-03	4.97	57.6	7.41	14,100	4.7
LP-04	6.07	59.47	8.04	19,200	7.9
LP-05	6.71	57.82	8.25	25,100	11.6
LP-06	7.63	49.82	8.19	43,100	22.8
LP-07	7.80	56.62	8.36	49,700	26.9
LP-08 ^2^	7.07	50.77	8.22	30,100	14.7
LP-09 ^2^	6.55	46.86	8.07	23,500	10.6
LP-10	5.24	52.85	6.74	12,400	23.9
LP-11	7.20	57.42	8.02	26,600	71.2
LP-12	7.28	58.76	8.79	36,100	13.6
LP-13	8.19	56.69	9.21	80,800	34.5
LP-14 ^3^	6.05	55.16	7.79	21,700	5.5
LP-15 ^3^	7.84	58.98	9.16	58,300	19.6
LP-16 ^3^	8.18	58.97	10.27	85,500	30.1
LP-17	8.18	59.09	9.30	95,800	21.1
LP-18	8.34	58.94	10.81	146,200	23.3
LP-19 ^2^	7.06	47.27	8.39	45,500	6.0
LP-20	7.57	59.28	8.67	39,700	28.6

^1^ The number average molecular weight (Mn) of copolymer LP was calculated from the content of nitrogen (N%) determined by elemental analysis and the Mn of macroinitiator LnMI. The average number of DMAEMA unit per arm, also called average degree of polymerization (DP) was calculated from Mn of copolymer LP and the average number of initiation site per LnMI macromolecule. ^2^ LP-08, 09 and 19 were treated with dilute HCl aqueous solution (0.1 M) to further improve their water solubility. ^3^ Data for LP-14,15 and 16 were reported previously [18].

## Data Availability

Data are available from the authors.

## Supplementary Materials

The following are available online at https://www.mdpi.com/article/10.3390/biom12010102/s1, Experimental Section for general characterization methods, dynamic light scattering and zeta-potential measurements, plasmid preparation, cells and media, gel retardation assay and cell viability assay.

## Sample Availability

Samples of the compounds are available from the authors.
