# Supplementary material for: Lignin-Based Nonviral Gene Carriers Functionalized by Poly[2-(Dimethylamino)ethyl Methacrylate]: Effect of Grafting Degree and Cationic Chain Length on Transfection Efficiency
Source: Biomolecules. 2022 Jan 8;12(1):102. doi: 10.3390/biom12010102 (PMC8773503; doi:10.3390/biom12010102)
Supplement: Supplementary file 1 [file biomolecules-12-00102-s001.zip › biomolecules-1519499-supplementary.pdf]

Supporting Information

Lignin-Based Nonviral Gene Carriers Functionalized by Poly[2-(dimethylamino)ethyl methacrylate]: Effect of Grafting Degree and Cationic Chain Length on Transfection Efficiency

Xiaohong Liu [1,2,†], Hui Yin [1,†], Xia Song [1], Zhongxing Zhang [1,*] and Jun Li [1,2,*]

[1] Department of Biomedical Engineering, National University of Singapore, 15 Kent Ridge Crescent, Singapore 119276, Singapore

[2] National University of Singapore (Chongqing) Research Institute, 2 Huizhu Road, Yubei District, Chongqing 401120, China

* Correspondence: biezhozh@nus.edu.sg (Z.Z.); jun-li@nus.edu.sg (J.L.)

† These authors contribute equally.

1. General Characterization Methods

The spectra of proton nuclear magnetic resonance (^1H NMR) were recorded on a Bruker AV-400 NMR spectrometer at 400 MHz at room temperature. The ^1H NMR measurements were carried out with an acquisition time of 3.2 s, a pulse repetition time of 2.0 s, a 30° pulse width, 5208 Hz spectral width, and 32 K data points. Chemical shifts were reported in ppm and referenced to the residual solvent peak (δ = 4.70 ppm for D_2O and δ = 2.50 ppm for DMSO-d_6). The elemental compositions of lignin-based macroinitiators and copolymers were tested by using a carbon, hydrogen, nitrogen, and sulfur (CHNS) instrument (Flash EA 1112, CE Instruments, ThermoFisher Scientific). Around 2 mg of dried samples were used for test in automatic mode.

2. Dynamic Light Scattering and Zeta-potential Measurements

Measurements of particle size and zeta potential of the complexes formed between cationic copolymers and pDNA were performed using a Zetasizer Nano ZS (Malvern Instruments, Southborough, MA, USA) with a laser light wavelength of 633 nm at a 173° scattering angle. Complex solutions (100 μL) containing 3 μg of pDNA (pRL-CMV) were prepared at various N/P ratios ranging from 2 to 30. The mixture was vortexed for 20 s, incubated for 30 min at room temperature and diluted in 1mL distilled water before analysis using the Zetasizer. The size measurement was performed at 25 °C in triplicate. The deconvolution of the measured correlation curve to an intensity size distribution was accomplished using a nonnegative least squares algorithm. The Z-average hydrodynamic diameters of the particles were given by the instrument. The Z-average size is the intensity weighted mean diameter derived from a cumulants or single-exponential fit of the intensity autocorrelation function. The zeta potential measurements were performed using a capillary zeta potential cell in automatic mode.

3. Plasmid Preparation

The plasmid used in this work was pRL-CMV (Promega, USA), encoding Renilla luciferase, which was originally cloned from the marine organism Renilla reniformis. The plasmid DNA was amplified in E. coli and purified according to the supplier's protocol (Qiagen, Hilden, Germany). The quantity and quality of the purified plasmid DNA was assessed by optical density at 260 and 280 nm and by electrophoresis in 1% agarose gel. The purified plasmid DNA was resuspended in TE buffer (10mM Tris-Cl, pH 7.5, 1mM EDTA) and kept in aliquots at a concentration of 0.5 mg/mL.

4. Cells and Media

All cell lines were purchased from ATCC (Rockville, MD). Hela, COS7 and MDA-MB-231 (human breast cancer cells) cells were maintained in Dulbecco's Modified Eagle's Medium (DMEM) supplemented with 10% heat-inactivated fetal bovine serum, 100 units/mg penicillin, 100 μg/mL

streptomycin at 37 °C and 5% CO_2. Opti-MEM reduced serum medium was purchased from Gibco BRL (Gaithersburg, MD).

5. Gel Retardation Assay

The binding ability of Lignin-PDMAEMA copolymers to pDNA was examined by gel electrophoresis. After adding 10 × loading buffer with polyplex solutions, the samples were loaded on a 0.8% agarose gel stained with 0.5 mg/mL ethidium bromide. The gels were run in 1 × TAE buffer (40 mM Tris-acetate, 1 mM EDTA) at 100 V for 30 min in a Sub-Cell system (Bio-Rad Laboratories, CA). DNA bands were visualized with a UV lamp on GelDoc system (Synoptics Ltd., UK).

6. Cell Viability Assay

Hela, COS7 and MDA-MB-231 cells were cultured in DMEM medium supplemented with 10% FBS at 37 °C, 5% CO_2, and 95% relative humidity. For cell viability assay, the cells with concentration of 10,000 cells/well were seeded into 96-well microtiter plates (Nunc, Wiesbaden, Germany). After 24 h, culture media were replaced with serum-supplemented culture media containing serial dilutions of sample and the cells were incubated for 24 h. Then 10 μL sterile filtered MTT (5 mg/mL) stock solution in PBS was added to each well, reaching a final concentration of 0.5 mg MTT/mL. After 5 h, unreacted dye was removed by aspiration. The formazan crystals were dissolved in 100 μL/well DMSO and the absorbance was measured by using a microplate reader (Spectra Plus, TECAN) at a wavelength of 570 nm. Six wells were treated together as a group. The relative cell growth (%) related to control cells cultured in media without lignin-PDMAEMA was calculated by $[A]_{test}/[A]_{control} \times 100\%$.
